# Uso de pantallas, sedentarismo y actividad física en los niños menores de seis años, durante el periodo de aislamiento social preventivo y obligatorio en AMBA: encuesta en línea

**DOI:** 10.31053/1853.0605.v80.n4.40343

**Published:** 2023-12-26

**Authors:** Sandra Viviana García, María Celeste Velazquez, Alberto Emiliano D´Agostino, Diego Julián Salto, Fernanda Melina Lardies Arenas, Sílvia Veronica Cuozzo, Ladislao Pablo Matias Diaz Ballve, Tatiana Dias de Carvalho

**Affiliations:** 1 Universidad Nacional de La Matanza. Departamento de Ciencias de la Salud, Kinesiología y Fisiatría Buenos Aires Argentina

**Keywords:** encuestas y cuestionarios, pandemias, preescolar, tiempo de pantalla, salud pública, surveys and questionnaires: pandemics, preschool, screen time, public health, enquetes e questionários, pandemias, pré-escola, tempo de tela, saúde pública

## Abstract

**Introducción:**

Dentro del contexto de la pandemia de COVID-19, los niños se quedaron confinados en sus hogares. La región del Área Metropolitana de Buenos Aires (AMBA) concentra la mayor densidad urbana de la Argentina y ha sido el epicentro de contagios y muertes por COVID-19. Los objetivos del presente estudio son caracterizar los hábitos del uso de pantalla, de sedentarismo y de la actividad física y describir sus asociaciones, en niños menores a seis años, del AMBA, Argentina.

**Métodos:**

estudio analítico y transversal, en que se utilizaron los datos de la encuesta en línea (Google Forms®). Mediante un muestreo no probabilístico por conveniencia, se invitaron a participar padres, madres, tutores legales y cuidadores de niños/as menores de seis años. La encuesta estuvo disponible durante cuatro meses del periodo de cuarentena y estuvo conformada por 31 preguntas sobre uso de pantalla, actividad física y sedentarismo de los niños.

**Resultados:**

fueron respondidas 256 encuestas, la mayoría (79,6%) era del género femenino y 50,8% en el rango etario de 31-40 años.

**Conclusión:**

La televisión es la más utilizada y los niños empiezan a usar pantallas táctiles a partir de un año. El tiempo de juego de los adultos con los niños y el tiempo que ellos suelen realizar actividades como leer un libro o dibujar es de una a dos horas por día. Hubo asociación entre ser dueño de la pantalla y usarla por más horas. Cuanto menor el nivel de ingreso, más tiempo los adultos juegan con sus niños.

## Abstract (en)

**Introduction:**

Within the context of the COVID-19 pandemic, children were confined to their homes. The Buenos Aires Metropolitan Area (AMBA) region has the highest urban density in Argentina and has been the epicenter of COVID-19 infections and deaths. The objectives of this study are to characterize the habits of screen use, sedentary lifestyle and physical activity and describe their associations, in children under six years of age from AMBA, Argentina.

**Methods:**

analytical and cross-sectional study, in which data from the online survey (Google Forms®) were used. Through a non-probabilistic convenience sampling, fathers, mothers, legal guardians, and caregivers of children under six years of age were invited to participate. The survey was available during four months of the quarantine period and was made up of 31 questions about screen use, physical activity, and sedentary lifestyle of children.

**Results:**

256 surveys were answered, the majority (79.6%) were female and 50.8% in the age range of 31-40 years.

**Conclusion:**

The television is the most used and children begin to use touch screens from one year. The play time of adults with children and the time that they usually carry out activities such as reading a book or drawing is one to two hours per day. There was an association between owning the screen and using it for more hours. The lower the income level, the more time adults play with their children.

## Resumo

**Introdução:**

No contexto da pandemia da COVID-19, as crianças ficaram confinadas em suas casas. A região da Área Metropolitana de Buenos Aires (AMBA) tem a maior densidade urbana da Argentina e foi o epicentro de infecções e mortes por COVID-19. Os objetivos deste estudo são caracterizar os hábitos de uso de tela, sedentarismo e atividade física e descrever suas associações em crianças menores de seis anos, de AMBA, Argentina.

**Métodos:**

: estudo analítico e transversal, no qual foram utilizados dados do questionário online (Google Forms®). Por meio de uma amostragem não probabilística por conveniência, pais, mães, responsáveis ​​legais e cuidadores de crianças menores de seis anos foram convidados a participar. A pesquisa ficou disponível durante quatro meses do período de quarentena e foi composta por 31 perguntas sobre uso de tela, atividade física e sedentarismo das crianças.

**Resultados:**

foram respondidos 256 inquéritos, sendo a maioria (79,6%) do sexo feminino e 50,8% na faixa etária de 31 a 40 anos.

**Conclusão:**

A televisão é a mais utilizada e as crianças começam a utilizar telas táteis a partir de um ano. O tempo que os adultos brincam com as crianças e que costumam fazer atividades como ler um livro ou desenhar é de uma a duas horas por dia. Houve associação entre possuir a tela e usá-la por mais horas. Quanto menor o nível de renda, mais tempo os adultos brincam com seus filhos.

**CONCEPTOS CLAVE**

**Que se sabe sobre el tema**

- Existe creciente preocupación por el aumento de la práctica de las pantallas, desde edades tempranas, que conlleva a retraso en habilidades cognitivas, lingüísticas, psicosociales y menor actividad física (mayor vida sedentaria), influyendo también sobre otros aspectos de su salud (sobrepeso y alteraciones en el sueño).
- Las directrices actuales recomiendan minimizar el tiempo de exposición a las pantallas en los niños pequeños.

**Que aporta este trabajo**

- El tipo de pantalla más utilizada es la televisión.
- Los/as niños/as empiezan a usar pantallas táctiles a partir de un año.
- Razones del uso de pantallas: entretener a los niños/as, realizar videollamadas con familiares y permitir que sus cuidadores realicen tareas.
- 1 a 2 horas/día es el tiempo de juego de los adultos con los niños y el tiempo que ellos suelen realizar actividades como dibujar.
- Cuando los niños son dueños de la pantalla la usan por más horas.
- Cuanto menor el nivel del ingreso, más tiempo los padres/tutores juegan con sus niños.

**Divulgación**

Desde muy pequeños, los niños ya utilizan las pantallas electrónicas, como televisión, tabletas y celulares inteligentes. Este estudio muestra que, durante la cuarentena, la televisión fue la pantalla más usada y que los adultos juegan con los niños y/o suelen realizar actividades como dibujar (o sea, sin las pantallas) entre 1 a 2 horas/día.

## Introducción

Los niños de hoy en día son, indudablemente, nativos digitales ^1^ . Su entorno está saturado de dispositivos electrónicos implantándose, a edades cada vez más tempranas, la cultura de las pantallas. No obstante, la decisión de si un niño puede tener un dispositivo electrónico, cómo y cuándo puede usarlo, depende de los adultos. Los lactantes se encuentran en un período de gran plasticidad cerebral, todas las experiencias ejercen profundas influencias sobre el desarrollo social, cognitivo, emocional y motor ^2^ , siendo fundamental la estimulación que los padres puedan ofrecer en el hogar los primeros años de vida ^3^ .

Las directrices actuales recomiendan minimizar el tiempo de exposición a las pantallas en los niños pequeños: no al uso en menores de dos años, no más de una hora por día de dos a cinco años, mantener los horarios de comidas y antes de dormir libres de pantallas; asegurar que el sedentarismo no sea parte de la rutina de los niños. Además, recomiendan a los padres una presencia activa, priorizando los contenidos educativos y adecuados a la edad ^4^ . En Argentina, existe discordancia entre las recomendaciones y el uso real de las pantallas en los niños pequeños. Se necesitan evidencias sobre los impactos, a largo plazo, del uso de la tecnología digital que permita, a los pediatras, un apoyo adecuado a las familias ^5^ .

La región del Área Metropolitana de Buenos Aires (AMBA) concentra la mayor densidad urbana de la Argentina, con más de un tercio de la población total del país. La alta aglomeración hace que la zona se convirtiera en el epicentro de contagios y muertes de coronavirus COVID-19 a nivel nacional ^6^ . En consecuencia, este territorio ha sufrido prolongación del período de aislamiento y exigencia en sus controles ^7^ , hecho que implica que los niños debieran sobrellevar mayor tiempo de confinamiento.

Existe creciente preocupación por el aumento de la práctica de las pantallas, desde edades tempranas, que conlleva a retraso en habilidades cognitivas, lingüísticas, psicosociales ^8-10^ y menor actividad física (mayor vida sedentaria), influyendo también sobre otros aspectos de su salud (sobrepeso y alteraciones en el sueño) ^11^ . Por lo tanto, los objetivos del presente estudio son caracterizar los hábitos del uso de pantalla, de sedentarismo y de la actividad física y describir sus asociaciones, en niños/as menores de seis años, del AMBA, Argentina.

## Materiales y Métodos

### Diseño y aspectos éticos

Fue realizado un estudio observacional, transversal y analítico, por medio de una encuesta anónima en línea. Todas las normas éticas internacionales de investigaciones en humanos, según la Declaración de Helsinki, así como normas nacionales de protección de pacientes ^12^ y de datos personales ^13^ fueron atendidas. El estudio recibió aprobación del comité de ética en investigación del Hospital Nacional Profesor Alejandro Posadas (394 EUPeS0/20) y todos los participantes firmaron el consentimiento informado.

### Población y criterios de elegibilidad

Mediante un muestreo no probabilístico por conveniencia, los sujetos fueron invitados a participar del estudio por medio de los enlaces para acceder la encuesta vía online mediante la herramienta Google Forms®, enviados por correo electrónico y por redes sociales.

Se incluyeron a todos los padres, madres, tutores legales y cuidadores de niños/as que accedieron a la encuesta, que aceptaron el consentimiento por internet, señalando la opción "sí" y que respondieron tener a su cargo niños menores de seis años, también señalando la respuesta "sí". Se excluyeron a los no residentes en la región del AMBA y aquellas encuestas incompletas.

### Prueba piloto

Previamente al inicio del estudio, y con el objetivo de determinar la viabilidad y comprensión de la encuesta, se llevó a cabo una prueba piloto a treinta participantes, donde se registró el tiempo requerido en responder y se realizaron preguntas específicas sobre la característica de la encuesta (dificultades que hubieran encontrado al contestar y sugerencias).

Como resultado, el tiempo promedio de los participantes fue insertado en el consentimiento informado (doce minutos) y se incluyeron algunas opciones de respuestas en la versión final de la encuesta, por ejemplo: sobre el uso de pantalla, se agregaron opciones relacionadas a si la usaban para realizar videollamadas, para actividades escolares, para telerehabilitación o teleasistencia médica. También se permitió la posibilidad de que los voluntarios respondiesen por un segundo hijo. No obstante, no fueron necesarios cambios en la cantidad o en la característica de las preguntas.

### Encuesta

La encuesta estuvo conformada por 31 preguntas, divididas en tres secciones (Material complementario). Luego del consentimiento informado, en la primera parte estaba la pregunta "¿ *Tiene niños menores de 06 (seis) años cumplidos a su cuidado?* ", la cual determinaría o no la continuación en la encuesta.

En la segunda parte, fueron recolectadas informaciones acerca de las/los voluntarios/as (municipio de residencia, género, edad, escolaridad, situación laboral, nivel de ingresos familiares, servicio de internet etc). En la última parte, fueron realizadas las preguntas sobre el uso de pantalla, actividad física y sedentarismo de los/as niños/as a su cuidado (tipos de dispositivos, tiempo de uso, finalidad y momentos de uso; así como tipo y tiempo en juegos y actividad física).

Treinta preguntas fueron cerradas ( *múltiple choice* ) y en algunas de ellas se utilizó la escala de Likert (Nunca, Casi nunca, Ocasionalmente, Casi siempre, Siempre). Solamente hubo una pregunta abierta, en que los/las participantes podrían agregar algún comentario. La encuesta estuvo disponible durante cuatro meses del periodo de aislamiento social.

### Variables de interés

Las preguntas de la encuesta contemplaron el uso de pantallas por los participantes y por los niños, el tiempo de actividad física y el tiempo de sedentarismo (con y sin pantallas), considerando las definiciones a continuación.

La actividad física representa el movimiento del cuerpo que usa energía por encima del consumo en estado de reposo. Según el grado de energía, que implique puede distinguirse: la ligera (caminar lentamente, bañarse, etc.) y de intensidad moderada a enérgica (caminar enérgicamente, correr en los juegos de pelota, etc.). Por su parte, el sedentarismo es definido como cualquier comportamiento en estado de vigilia caracterizado por un gasto energético de ≤1,5 MET, sea en posición sentada, reclinada o acostada ^14^ .

Por tiempo sedentario con pantallas - definidas como superficie con capacidad para emitir luz y formar imágenes - se considera el tiempo que permanecen ante ellas mirando pasivamente algún pasatiempo, sea televisión, computadora, consolas de videojuegos o dispositivos móviles. Mientras tanto, el tiempo dedicado a actividades sedentarias que no implican una pantalla hace referencia al tiempo que incluye permanecer tendido en una alfombra, sentado en una sillita alta para bebé o en el cochecito con poco movimiento, estar sentado leyendo un libro o jugando un juego tranquilo, entre otros de similares características ^14^ .

### Análisis estadístico

Las variables categóricas se reportaron como número de presentación y porcentaje. Para determinar la distribución de la muestra se utilizó la prueba de Kolmogorov-Smirnov. Las variables continuas que asumieron una distribución normal fueron reportadas como media y desvío estándar (DE). Las asociaciones entre las variables dependientes e independientes fueron realizadas por las pruebas de Chi-cuadrado de Pearson y Kruskal-Wallis. Para el análisis de los datos se utilizó el software IBM SPSS Macintosh, versión 24.0 (IBM Corp, Armonk, NY, USA).

## Resultados

Fueron respondidas 387 encuestas, de las cuales se excluyeron 131 por no tener hijos menores de seis años, por no vivir en la región del AMBA y por no completar todas las respuestas. De los encuestados, 79,6% era del género femenino y 50,8% en el rango etario de 31-40 años. Las demás características sociodemográficas de los participantes, así como sus hábitos de uso de pantallas, están presentadas en la Tabla 1.

**Tabla N° 1:** Características sociodemográficas y hábitos de uso de pantallas de los participantes.

	N=256	%
Género		
Femenino	203	79,6
Masculino	52	20,4
Edad		
Menor de 30 años	65	25,4
31 a 40 años	130	50,8
41 a 50 años	58	22,7
Mayor de 51 años	3	1,2
Residencia		
GBA zona oeste	141	55,1
CABA	55	21,5
GBA zona sur	41	16
Otro lugar de residencia	10	3,9
GBA zona norte	9	3,5
Instrucción		
Universitario completo	101	39,5
Universitario incompleto	54	21,1
Terciario completo	39	15,2
Secundario completo	31	12,1
Otros	31	12,1
Tipo de familia		
Una madre y un padre	204	79,7
Padres separados	30	11,7
Solo madre o padre	13	5,1
Otra	8	3,1
Homoparental	1	0,4
Principal fuente de ingreso		
Relación de dependencia	144	56,3
Trabaja en casa	128	50
Trabaja normalmente fuera de casa	45	17,6
Trabaja fuera de casa con horario reducido	37	14,5
No trabaja	28	10,9
Perdió la actividad laboral	17	6,6
Jubilado	1	0,4
Horas que trabaja por día		
Menos de 6 horas	99	38,7
6 a 8 horas	99	38,7
Más de 8 horas	58	22,7
Nivel de ingreso neto del último mes *		
Menos de 20.000 pesos	31	12,1
20.001 a 40.000 pesos	70	27,3
40.001 a 60.000 pesos	70	27,3
Superior a 60.000 pesos	85	33,2
Acceso a internet en el hogar		
Servicio de internet	241	94,1
Datos del celular	15	5,9
Utiliza pantallas electrónicas		
Siempre	149	58,2
Casi siempre	83	32,4
Ocasionalmente	23	9
Nunca	1	0,4
Tiempo que las utiliza por día		
1-3 horas	37	14,5
3-5 horas	79	30,9
5-8 horas	81	31,6
Más de 8 horas	58	22,7
Cantidad de niños/as menores de 6 años que tiene a cargo en ASPO		
1 niño/a	192	75
2 niños/as	56	21,9
3 o más niño/as	8	3,1
La vivienda posee espacio de recreación		
Si	139	54,3
No	117	45,7

* Rangos determinados considerando el valor de la canasta básica argentina en abril/2020. Argentina R. Valorización mensual de la canasta básica alimentaria y de la canasta básica total. Gran Buenos Aires. Condiciones de vida [Internet]. [citado 19/07/2022];4:17. Disponible en: https://www.indec.gob.ar/uploads/informesdeprensa/canasta/ 12_2034B9869B75.pdf

Con relación al perfil de los/as niños/as bajo su cuidado, 38,70% tenían 5 años, 96,50% no presentaban retraso en el desarrollo y 55,10% eran del género masculino.

La tabla 2 presenta las características del uso de las pantallas por los niños. El 62,1% afirman siempre supervisar lo que el niño/a mira; y 43 % ocasionalmente comparte con el niño el tiempo y la actividad frente a las pantallas.

**Tabla N° 2:** Características del uso de las pantallas por los niños

Tipos de pantalla	N=256	%		
Televisor + fijos y móviles	221	86,3		
Televisor + celular o tablets	32	12,5		
Televisor + computadoras o consolas	3	1,5		
Pantallas en el momento de irse a la cama				
Nunca	86	33,6		
Casi nunca	45	17,6		
Ocasionalmente	63	24,6		
Casi siempre	45	17,6		
Siempre	17	6,6		
Pantallas usadas en el momento de irse a la cama *				
Televisor	78	45,9		
Celular/Tablet	47	27,6		
Todas	30	17,7		
No utiliza	11	6,5		
Computadora/consola	4	2,4		
Actividades *sin* uso de pantalla/s				
Todas (comidas, baño, juegos)	202	78,9		
Siempre está usando	20	7,8		
Momento de juego	14	5,5		
Durante el baño	11	4,3		
Durante las comidas	9	3,5		
Motivos para brindar la pantalla				
Entretenimiento	162	63,3		
Videollamada con familiares	73	28,5		
Actividades educativas/deportivas o teleasistencia de salud	21	8,2		

* N=170

Las figuras 1 y 2 presentan la distribución de horas de uso según el tipo de pantalla y la edad en que comenzaron a realizar ciertas actividades con la misma, respectivamente. La figura 3 presenta la distribución entre la cantidad de horas durante el día en que el/la niño/a realiza juegos con actividad física.

**Figura N° 1.**
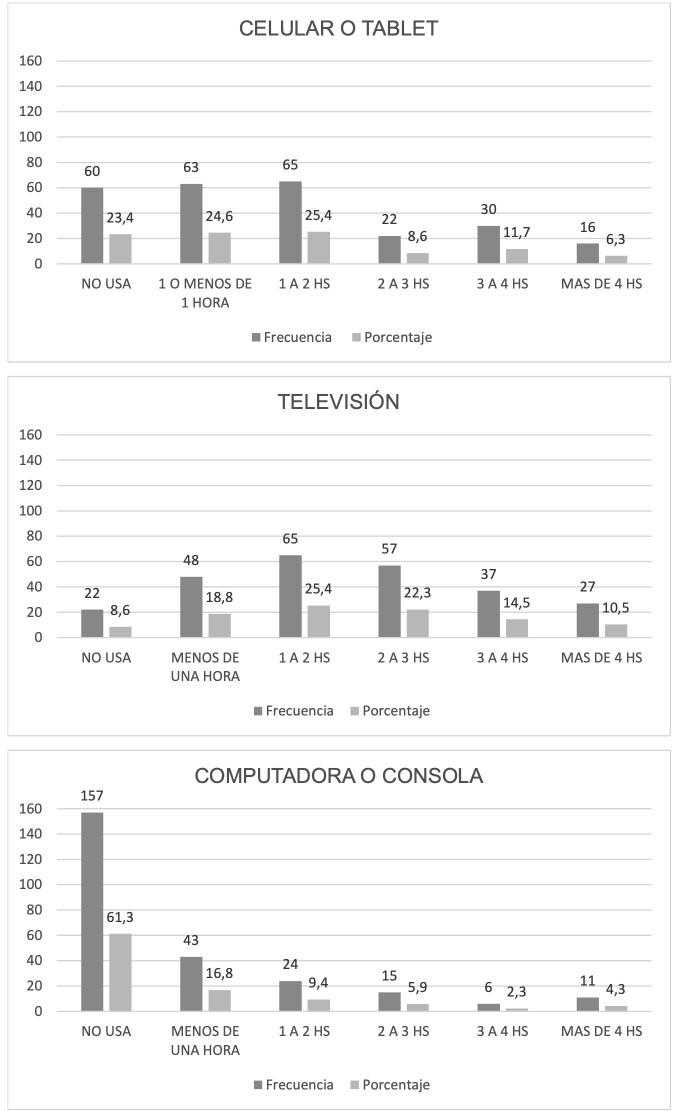
Horas diarias frente a las pantallas.

**Figura N° 2.**
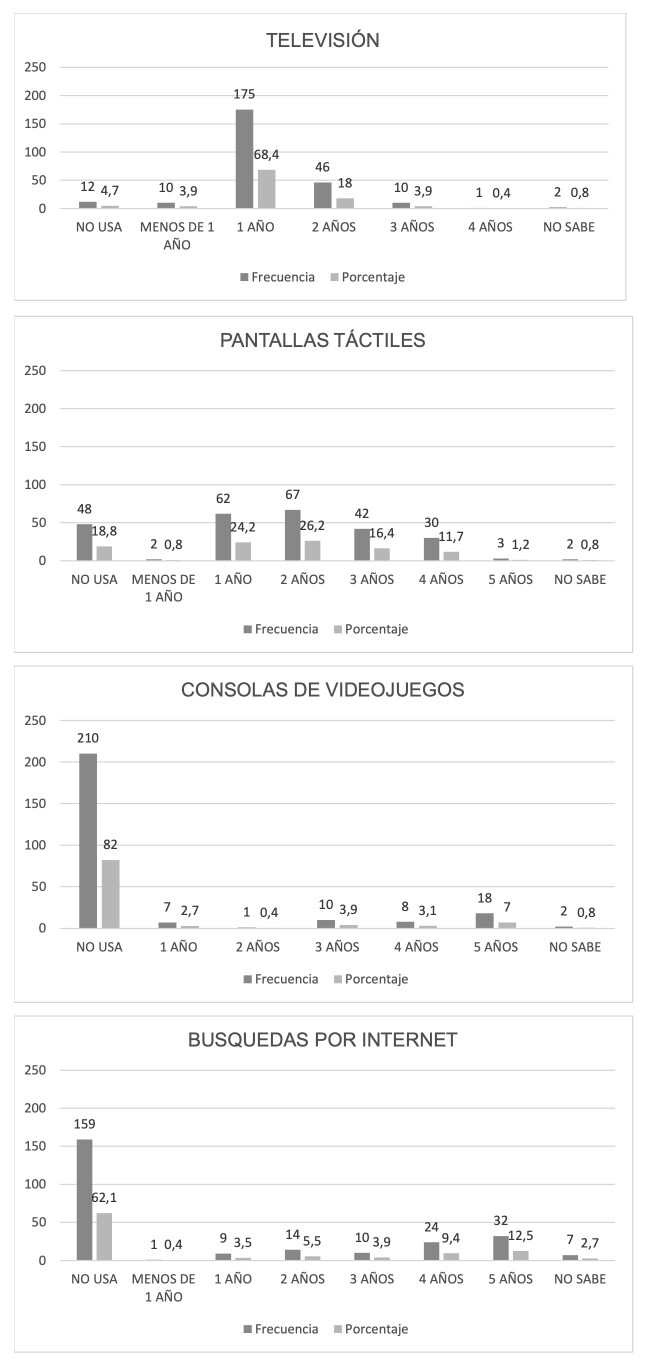
Edad del inicio del uso de diferentes tipos de pantallas.

**Figura N° 3.** Tiempo por día de juego con actividad física. A: Arrastrarse por el piso, caminar lentamente, hacer equilibrio. B: Gatear, caminar enérgicamente, trepar, correr, saltar, bailar, triciclo o bicicleta.

De los encuestados, 55,1% expresan conocer bastantes juegos que no estén relacionados con pantallas y 42,2% ocasionalmente lo ponen en práctica. La figura 4 expresa la cantidad de horas en el día en que el/la niño/a está sentado haciendo actividades sin una pantalla.

**Figura N° 4.**
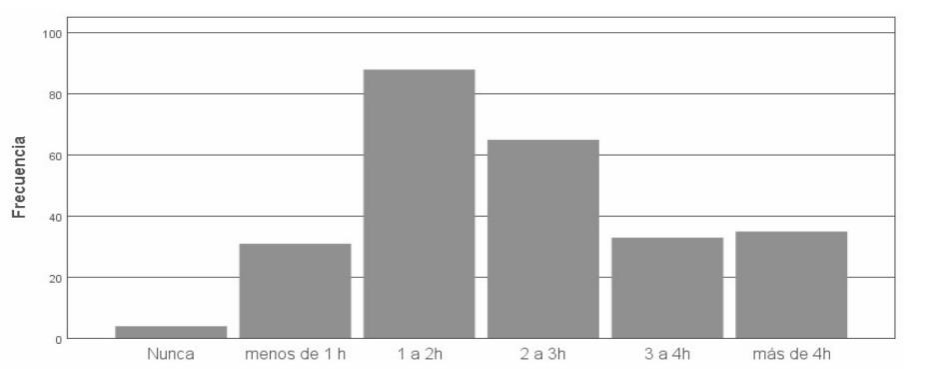
Tiempo por día sedentario sin uso de la pantalla.

De los encuestados, 46,9% afirman que ningún profesional le recomendó sobre los riesgos/beneficios de la utilización de pantallas y 62,9% considera inadecuado su uso en niños/as desde temprana edad.

Hubo asociación significativa entre más horas uso de pantalla y cuando esta pertenecía al niño. También encontramos asociación entre el nivel de ingreso y el hábito de jugar con el niño, cuanto menor el nivel de ingreso, más horas de juego con el niño.

## Discusión

Los principales hallazgos de este estudio indican que el tipo de pantalla más utilizada es la televisión y que los/as niños/as empiezan a usar pantallas táctiles a partir de un año. Las principales razones del uso de pantallas son: entretener a los niños/as, realizar videollamadas con familiares y permitir que sus cuidadores realicen tareas. El tiempo de juego de los adultos con los niños y el tiempo que ellos suelen realizar actividades como dibujar es de una a dos horas por día. Cuando los niños son dueños de la pantalla la usan por más horas y cuanto menor el nivel del ingreso, más tiempo los padres/tutores juegan con sus niños.

La mayoría de las encuestadas son mujeres y el nivel de estudios predominante es el universitario completo, al igual que una encuesta realizada en Chile ^15^ . También en comparación a dicho estudio, existieron datos similares en cuanto a disponer de espacios de recreación en el hogar, siendo mayoritariamente respuestas afirmativas; y en cuanto al acceso a internet, las viviendas cuentan con servicio de internet pago y, en menor medida, solamente con datos del celular.

Los resultados muestran que el tipo de pantalla más utilizada es la televisión. Al igual que en otros trabajos ^2,5, 16^ , la exposición a la televisión fue siempre mayor que otras actividades, seguida por las pantallas móviles. El uso del televisor se mantiene constante en todos los grupos etarios, sin embargo, al aumentar la edad, los dispositivos de preferencia son los móviles ^2^ . En la mayoría de las casas, la televisión suele estar encendida de fondo sumando una pantalla más. Esto provoca que su uso no sea considerado como una exposición, debido a que los tiempos de atención puesta en la misma (sobre todo en los niños más pequeños) son de corta duración o discontinuos. Esta situación podría afectar negativamente el uso y la adquisición del lenguaje, la atención, el desarrollo cognitivo y la función ejecutiva en niños menores de cinco años ^8, 17^ .

Asimismo, se constató que, aunque ocasionalmente, la mayoría de los encuestados utiliza alguna pantalla al momento de ir a la cama, siendo nuevamente el televisor el elegido por más de la mitad, seguido por las pantallas móviles. La evidencia ^8^ apoya el hecho de que la exposición a la luz (particularmente a la luz azul) y la actividad de las pantallas antes de acostarse afecta los niveles de melatonina pudiendo retrasar o alterar el sueño, perjudicando el rendimiento escolar y el comportamiento. Según los resultados de diecisiete países participantes del estudio SUNRISE ^18^ , con relevamiento especial en contexto de COVID-19, se notifica que el 64% de los niños usan dispositivos electrónicos en las dos horas antes de acostarse; el 32 % de los niños tienen acceso a un dispositivo de pantalla electrónica en la habitación donde duerme, con un promedio de tiempo de pantalla de 137,5 min por día.

Con respecto a la cantidad de horas frente a las pantallas, la mayor parte de los hallazgos exceden las recomendaciones ^14, 19^ . A esto se añade que los/as niños/as empiezan a usar las pantallas táctiles a partir del año de vida, incluso antes, aunque en menor porcentaje. Estudios previos a la pandemia revelaron resultados similares, el promedio de uso diario fue de 2,25 horas al día ^19^ y la edad de comienzo antes de un año ^19-20^ .

De manera significativa se pudo comprobar que cuando los niños/as son dueños/as de la pantalla mayor es su exposición, dedicando más horas a su uso y, aunque sin significancia, a mayor edad también mayor tiempo de uso. La Subcomisión de Tecnologías de Información y Comunicación de la Sociedad Argentina de Pediatría ^21^ y la OMS ^14^ desaconsejan el uso antes de los dieciocho meses de edad y recomiendan una absoluta supervisión entre los 18 y los 24 meses. Desde los dos años, el tiempo recomendado es de no más de sesenta minutos diarios y se agrega el acompañamiento para evitar exposición a contenidos inapropiados y en horarios que afecten el sueño y la comunicación familiar ^21^ .

A raíz de la pandemia por COVID-19, las directrices de límites basados en el tiempo fueron complementadas con la regla de las tres "C", *children, contents, context* (niño, contenido y contexto), priorizando la calidad del contenido y pautando su uso, sin ofrecer las pantallas como recompensa e interactuar con los/as niños/as durante su tiempo frente a ellas ^11,17, 19^ . Así pues, las medidas de confinamiento han provocado un aumento del tiempo de pantalla en muchas regiones. En España, dos de cada tres niños menores de cuatro años usaron teléfonos inteligentes y tabletas diariamente ^22^ .

En cuanto a la actividad física, el mayor porcentaje no cumplió con las pautas recomendadas de, al menos, 180 minutos de actividad diaria sin distinción de intensidad, dando como corolario un gran porcentaje de sedentarismo. No obstante, más de la mitad de los/as niños/as cumple con la recomendación de, al menos, sesenta minutos de actividad de intensidad moderada a vigorosa por día ^14^ . En entrevistas realizadas a padres de niños en edad preescolar de China ^23^ se encontró, en comparación a antes de la COVID-19, niveles de actividad física muy bajos por confinamiento de los niños/as que no podían salir al aire libre.

Del tiempo dedicado a actividades sedentarias que no implican una pantalla, resultó que 59,8% de los/as niños/as las realizaron por un periodo de una a tres horas por día y un 26,6% entre tres y más horas diarias. En este sentido, se aconseja que los/as niños/as no deberían permanecer inmovilizados más de una hora seguida. En los que no logran aún desplazamientos, deben incluir treinta minutos de posición prona mientras estén despiertos, repartidos a lo largo del día. En caso de estar quietos, se recomiendan actividades interactivas que no incluyan pantallas (narración de historias, etc.) ^14^ .

A pesar de que más de la mitad de los participantes consideran inadecuado el uso desde temprana edad y conocen bastantes juegos que no se relacionan con las pantallas, el entretenimiento resultó ser la mayor de las razones para el uso de pantallas. Es interesante advertir la forma en que se toma este uso, en reemplazo del juego tradicional como forma de entretenimiento. No obstante, afirman supervisar los contenidos un 62,1%. Otros estudios ^2,5,16-17, 20^ mencionan que se reduce la cantidad y la calidad de la interacción entre padres e hijos y distrae del juego. Interesantemente, en este estudio, se observó una asociación entre el nivel de ingreso y el hábito de jugar con el niño, a menor el nivel de ingreso, más horas de juego con el niño. Contrariamente, una cohorte de doce países ^22^ reveló que, en las familias de niveles socioeconómicos más bajos, durante el confinamiento, hubo un aumento del tiempo de exposición a pantallas, con un uso desde muy temprana edad.

La literatura ^2,8,16, 19^ demuestra que la exposición a las pantallas tiene un impacto negativo, generando riesgos en el desarrollo, siendo responsabilidad de los adultos otorgar permiso para su uso. De igual modo, la preocupación mundial radica en que, previo a la COVID-19, ya la quinta parte de los niños en edad preescolar no cumplían con las pautas de movimiento ^24^ y, de convertirse este comportamiento en la nueva normalidad, podría traer consecuencias de salud y económicas ^23^ . Es conveniente observar que aproximadamente la mitad de los/as encuestados/as afirma no haber recibido recomendaciones de profesionales en cuanto a riesgos/beneficios del uso de pantallas.

La principal limitación de este estudio es que, por tratarse de una encuesta en línea, puede haber cierta dificultad en las respuestas de aquellas personas que no son hábiles en el manejo de las nuevas tecnologías, desistiendo de terminar de responder. A pesar de esto, en el mejor de nuestro conocimiento, esta investigación contiene el mayor número de participantes en un estudio nacional, describiendo las características del uso de pantallas electrónicas en Argentina, durante el periodo de pandemia.

Considerando que la actividad física presenta beneficios como factor de prevención cardio metabólico y es el medio por el cual los niños desarrollan su aspecto psicomotor ^25^ , podría ser una posibilidad de investigación a futuro, además del enfoque en cantidad de horas de exposición a pantallas, observar el tiempo de actividad física, ya que no es lo mismo estar tiempo expuesto a pantallas con poco o sin tiempo de actividad física, que, aun teniendo exposición a pantallas, realizar actividad física. Adicionalmente, estudios futuros podrían comparar si las horas de uso de pantalla y/o el tiempo dedicado a la actividad física u otras actividades han cambiado después de finalizada la condición de cuarentena.

## Conclusiones

La televisión es el tipo de pantalla más utilizado y empiezan a utilizarla, junto a las táctiles, desde temprana edad. La mayor parte no cumple con las recomendaciones para la actividad física y comportamiento sedentario diarios. El tiempo mayor de uso se asoció a ser dueño de la pantalla. El tiempo de juego de los adultos con los niños y actividades sin pantallas es de una a dos horas por día. Hubo asociación entre menor nivel de ingreso y más tiempo en que los adultos juegan con sus niños. Estas informaciones podrán contribuir con el manejo de profesionales de salud, educación y gestión sobre del tema. Y, lo más importante, que los padres/madres/tutores puedan reflexionar sobre el uso más conveniente de esos recursos electrónicos, en el desarrollo de sus niños/as.
